# Retrospective meta-transcriptomic identification of severe dengue in a traveller returning from Africa to Sweden, 1990

**DOI:** 10.1016/j.onehlt.2021.100217

**Published:** 2021-01-22

**Authors:** Kristian Alfsnes, Nina Lagerqvist, Sirkka Vene, Jon Bohlin, Jenny Verner-Carlsson, David Ekqvist, Andreas Bråve, Edward C. Holmes, Weifeng Shi, John H.-O. Pettersson

**Affiliations:** a Infectious Disease Control and Environmental Health, Norwegian Institute of Public Health, Oslo, Norway; b Public Health Agency of Sweden, Nobels väg 18, SE-171 82 Solna, Sweden; c Department of Infectious Diseases, University Hospital Linköping, Sweden; d Marie Bashir Institute for Infectious Diseases and Biosecurity, School of Life and Environmental Sciences and School of Medical Sciences, the University of Sydney, Sydney, New South Wales 2006, Australia; e School of Public Health, Shandong First Medical University & Shandong Academy of Medical Sciences, Taian 271016, China; f Zoonosis Science Center, Department of Medical Biochemistry and Microbiology, Uppsala University, Sweden

**Keywords:** Dengue virus, Dengue haemorrhagic fever, Viral haemorrhagic fever, Meta-transcriptomics, RNA-sequencing, Human pegivirus

## Abstract

Pathogens associated with haemorrhagic fever commonly have zoonotic origins. The first documented imported case of likely viral severe haemorrhagic fever in Sweden occurred in 1990. Despite extensive study, no aetiological agent was identified. Following retrospective investigation with total RNA-sequencing of samples collected between 7 and 36 days from onset of symptoms we identified dengue virus 3 (DENV-3) and a human pegivirus (HPgV). We conclude that the patient likely suffered from haemorrhagic symptoms due to an atypical severe and undiagnosed dengue infection.

## 1 Background

More than 20 enveloped RNA viruses are known to cause haemorrhagic fever, largely from four groups - arenaviruses, filoviruses, bunyaviruses, and flaviviruses [1], the majority of which originate from arthropod vectors and vertebrate animal sources and hence are of major importance in a One Health context [2]. These pathogens can induce symptoms ranging from asymptomatic to severe life-threatening conditions, with some haemorrhagic fevers associated with mortality rates as high as 80% [1]. A major complicating factor is that the initial clinical presentations commonly overlap with syndromes that have a less severe clinical course. A rapid diagnosis is therefore of major importance.

We utilized total RNA-sequencing (‘meta-transcriptomics’) to investigate Sweden's first reported case of imported haemorrhagic fever for which the aetiological agent was never identified. The background, initial handling and laboratory analyses, clinical data, epidemiology, and outcome have been described previously [3,4]. Briefly, a male Swedish traveller returned from a three-month visit in central and eastern Africa in January 1990, the last month of which was spent in Kenya where he was bitten by mosquitoes and bed-bugs and also came in contact with monkeys. Five days after arriving in Sweden, the patient fell ill with febrile symptoms. On the fourth day after onset of symptoms, the patient's condition deteriorated rapidly, whereafter he was admitted to a hospital and shortly thereafter moved to an intensive care unit. Soon after transferal to the intensive care unit, symptoms expanded to include internal haemorrhaging. During the haemorrhagic episode, which began at the start of week two and ceased at the end of the third week after symptom onset, the patient had received 65 units of blood. It is noteworthy that the patient had an extensive febrile period, with temperatures consistently above 40 °C, that lasted for approximately three weeks despite symptomatic treatment. His clinical course also included sepsis, respiratory failure and disseminated intravascular coagulation with thrombocytopenia. After 4.5 weeks, the patient's condition improved and he was moved from the intensive care unit to a regular ward. He was discharged 2.5 months after onset of symptoms.

Initial differential diagnoses excluded leptospirosis, malaria, rickettsiosis, typhoid fever and septicaemia. Despite attempts with cell-, rodent- and primate inoculation, serological tests, blood smears and electron microscopy, no aetiological agent was identified and no definitive diagnosis was reached. It is noteworthy that PCR was not available at this point of time. However, it was suggested that the haemorrhagic fever might be due to a novel virus, potentially an undescribed filovirus as indicated by electron microscopy images taken during the first week of symptoms [3].

## 2 Methods

We performed a retrospective meta-transcriptomic investigation using a set of separately aliquoted −80 °C stored plasma and urine samples collected between 7 and 18 days and 28–36 days after onset of symptoms, respectively (Table 1). Oral and written informed consent was obtained from the patient. This approach allows for a sensitive and unbiased identification and characterization of all RNA transcripts in a particular sample with the potential to identify both known and novel pathogens [5]. The methodology used herein has been described previously [5,6]. Briefly, total RNA was extracted using a combination of trizol–chloroform separation and automated magnetic bead total nucleic acid extraction using a MagLEAD system (Precision System Science Co.). Following ribosomal RNA depletion using the Ribo-Zero Gold Kit (Illumina), paired-end (100 bp read-length) libraries where created with (i) the TruSeq stranded TotalRNA kit (Illumina) and sequenced on an Illumina NovaSeq 6000 instrument at the Science for Life Laboratory, Uppsala, Sweden, and (ii) Trio RNA-seq library preparation kit (NuGen) and sequenced on the BGI BGI500 (paired-end, 100 bp read-length) platform at BGI, Hong Kong. Subsequently, all RNA sequence libraries were quality trimmed with Trimmomatic v.0.36 (www.usadellab.org/cms/?page=trimmomatic) and human reads were removed using bbmap v.3832 (https://jgi.doe.gov/data-and-tools/bbtools/) and the human reference genome (RefSeq assembly: GCF_000001405.38). The quality-passed and human-trimmed reads were then screened with Diamond v.0.9.15.116 (https://github.com/bbuchfink/diamond) and blastn against the complete non-redundant protein (nr) and nucleotide (nt) databases, respectively.

**Table 1:** RNA-seq, PCR and serological results for each sample.

	**Days from onset**⁎					**DENV**			**HPgV**		
**Sampling date**		**Sample ID**	**Material**	**RNAseq**	**# of reads**	**PCR**	**IgM**	**IgG**	**RNAseq**	**# of reads**	**PCR**
1990-01-24	7	OA	Plasma	+	57	+			+	53	−
1990-01-24	7	OB	Plasma	+	14	+			−	0	+
1990-01-27	10	M	⁎⁎			+	+	≥2560			
1990-01-28	10	L	⁎⁎			+	+	≥2560			
1990-01-30	14	1B	Plasma	+	30	+			+	8	+
1990-01-30	14	1C	Plasma	+	24				+	38	
1990-01-30	14	N	⁎⁎			+	+	≥2560			
1990-01-31	15	2A	Plasma	+	32				+	8	
1990-01-31	15	2B	Plasma	+	18	+			+	8	+
1990-02-01	16	3	Plasma	+	124				−	0	
1990-02-02	17	4A	Plasma	+	213				−	0	
1990-02-02	17	4B	Plasma			+					−
1990-02-04	19	5A	Plasma	+	80				−	0	
1990-02-04	19	5B	Plasma	+	30	+			−	0	−
1990-02-04	19	5C	Plasma	+	146				−	0	
1990-02-14	29	6	Urine	−	0	−			−	0	
1990-02-18	33	7	Urine	−	0	−			−	0	
1990-02-18	33	8	Urine	−	0				−	0	
1990-02-22	37	9	Urine	−	0	−			−	0	

⁎ Onset of symptoms = 1990-01-17.

⁎⁎ Plasma or serum.

## 3 Results

Following inspection of the read hits to known viruses, we were able to identify sequence reads with high sequence similarity to dengue virus (DENV) in 11 of 15 sequencing libraries. No other potential pathogens were identified, aside from human pegivirus (HPgV), formerly known as GB virus C, which is usually associated with benign infections [7], in five of the libraries (Table 1).

All reads of DENV origin were retrieved and re-screened on NCBI blast to identify the most similar reference genome. Accordingly, a complete DENV-3 genome collected 1985 in Mozambique (GenBank accession FJ882575.1) was found to exhibit the greatest sequence similarity to the DENV reads present in the patient libraries. Subsequently, all individual sequence libraries were mapped against the DENV-3 reference genome from Mozambique using bbmap. Following the merger of read mapping files this resulted in a total of 768 reads (including duplicate reads) covering approximately 38% (4055 of 10,562 bp) of the viral genome. Similarly, all sequence libraries were mapped against a HPgV reference genome from USA (MK291245.1) using bbmap, resulting in a total of 115 reads (including duplicate reads) for all HPgV positive libraries covering ~18% (1641 of 9319 bp) of the reference genome.

**Fig. 1:**
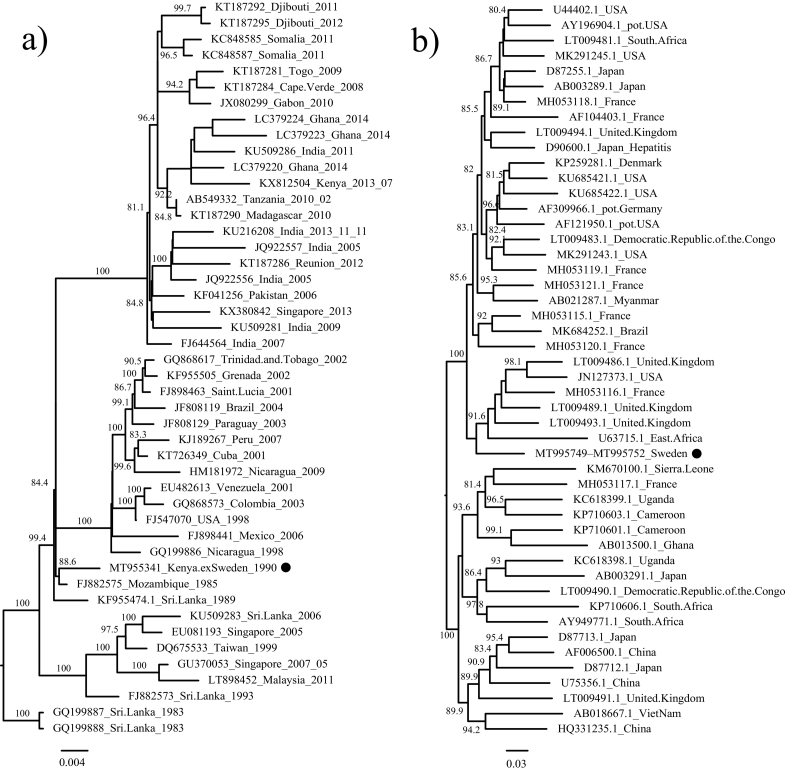
Phylogenetic trees of (a) DENV-3 and (b) HPgV, respectively, with representative sequences added to show evolutionary context. The tree was mid-point rooted for visual purposes only. Numbers on branches indicate bootstrap support above 80%. Scale-bars indicates the number of nucleotide substitutions per site.

To place the partial DENV-3 genome in an evolutionary and epidemiological context, closely related sequences were retrieved from NCBI/GenBank and aligned using Mafft v.7.271 (https://mafft.cbrc.jp/alignment/software/) employing the *E*-INS-i algorithm. A maximum likelihood tree was then inferred, via IQ-TREE v.1.6.11 [8], using the TIM2 + F + Γ_4_ model of nucleotide substitution and a Shimodaira–Hasegawa-like test to assess branch support. The resultant tree was viewed, annotated in FigTree v.1.44 (https://github.com/rambaut/figtree) and mid-point rooted. The DENV-3 sequence identified here shared a most recent common ancestor (88.6% bootstrap support) with the reference sequence from Mozambique, compatible with an African origin for the Swedish infection (Fig. 1). The same analytical procedure was performed HPgV using the GTR + F + R6 model of nucleotide evolution. The partial HPgV sequence was found within a group of viruses of diverse geographic origins, although including east Africa (81.5% bootstrap support) (Fig. 1).

Importantly, the results of the meta-transcriptomic analysis were confirmed with real-time PCR, commercial ELISA (Dengue Virus IgM Capture DxSelect, Focus Diagnostics), and immunofluorescence analysis detecting dengue virus IgG antibodies [9] (Table 1). Convalescent sera were not available for analysis. Although negative results can never definitively exclude an infection of a different aetiological agent, all samples that tested positive for DENV with real-time PCR [10] also tested negative for Ebola virus, Crimean-Congo Haemorrhagic Fever virus, Marburg virus, Rift Valley fever virus, and Lassa virus using PCR (Supplementary Table S1).

All sequence libraries generated in this project, in which human reads have been removed, have been deposited at the NCBI Short Read Archive under BioProject ID: PRJNA661184. The partial DENV-3 and HPgV consensus genomes generated have been deposited in NCBI GenBank (accession numbers: MT955341 [DENV3]), MT995749–MT995752 [HPgV]).

## 4 Discussion

In early 1990, a traveller returned to Sweden following travel to central and eastern Africa and developed an undiagnosed severe haemorrhagic fever. Based on the clinical data reported for this patient [3,4] and the meta-transcriptomic identification of DENV-3 that was confirmed by a variety of other assays, we suggest that the symptoms (see also supplementary Table S2), including protracted febrile illness and prolonged viraemia, likely represent clinical manifestations of an atypical severe dengue haemorrhagic fever [11].

Over the last 50 years the four mosquito-borne DENVs (DENV-1 to DENV-4) have spread globally and become established in almost all regions, where previously only one serotype was present. Although DENV is likely endemic in many African countries, infections with this virus have only been reported sporadically from African countries [12]. Larger epidemics, of endemic and sylvatic DENV (i.e. involving both non-human primates and mosquitoes in rural environments) have been reported for DENV-1 and DENV- 2 since the late 1970s, such as in the Seychelles and Kenya providing examples, [13], with DENV-3 first being identified in Mozambique in 1984 [14]. Phylogenetic analysis shows that the DENV-3 identified here groups with the sequence from Mozambique from the 1984–1985 epidemic, compatible with an African origin (Fig. 1). This adds support to the conclusion that the patient was infected with DENV-3, most likely during his final one-month stay in Kenya, a location where DENV is greatly under-sampled and -diagnosed. A DENV-3 infection might also explain why there were no secondary cases to any of the care givers or laboratory staff at the hospital, although direct blood exposure was reported [3].

In five of the sequencing libraries (Table 1), partly confirmed with PCR, we noted the presence of HPgV, although of uncertain geographic origin (Fig. 1). HPgV is a common virus, present in up to 5% of healthy blood-donors [7], and is most closely related to simian pegiviruses [15]. HPgV is currently not definitively associated with any disease [16], and the majority of individuals clear the infection within two years [7]. However, HPgV co-infection has been linked to increased survival rates in patients infected with Ebola virus and human immunodeficiency virus [17,18]. Although HPgV was found to be present before and during the haemorrhagic episode in the present study, we cannot draw any conclusions as to its potential clinical impact.

In conclusion, we show that total RNA-sequencing is powerful tool to investigate undiagnosed cases caused by an infectious aetiological agent, with the potential to identify possible viral co-infections and sporadic zoonotic infections, and so enhancing One Health surveillance and diagnostic programs. Our study also adds support to the notion that dengue virus has likely been present in many sub-Saharan Africa for decades, possibly causing unrecognized cases of dengue fever and more severe dengue disease, including that with atypical symptoms as documented here. Increased surveillance efforts will aid in more accurate estimation of the true burden of dengue in sub-Saharan African countries [19].

The following are the supplementary data related to this article.

Supplementary Table S1 Primer and probe information for PCR methods. Supplementary Table S1

Supplementary Table S2 Summary of clinical symptoms and findings. Supplementary Table S2

## Author statement

Kristian Alfsnes: Formal analysis, Roles/Writing - original draft.

Nina Lagerqvist: Investigation.

Sirkka Vene: Investigation.

Jon Bohlin: Formal analysis.

Jenny Verner-Carlsson: Investigation.

David Ekqvist: Investigation.

Andreas Bråve: Investigation.

Edward C. Holmes: Funding acquisition, Writing - review & editing.

Weifeng Shi: Conceptualization, Funding acquisition.

John H.-O. Pettersson: Conceptualization, Formal analysis, Funding acquisition, Roles/Writing -.

original draft.

## Declaration of Competing Interest

All authors have read the manuscript and have no conflict of interest relating to the manuscript.
