# Supplementary material for: Retrospective meta-transcriptomic identification of severe dengue in a traveller returning from Africa to Sweden, 1990
Source: One Health. 2021 Jan 22;12:100217. doi: 10.1016/j.onehlt.2021.100217 (PMC7851179; doi:10.1016/j.onehlt.2021.100217)
Supplement: Supplementary Table S1 — Primer and probe information for PCR methods. [file mmc1.docx]

Supplementary table S1. Primer and probe information for PCR methods.

| **Pathogen** | **Target** | **Probe** | **Forward** | **Reverse** | **Ref** |
| --- | --- | --- | --- | --- | --- |
| Crimean Congo Haemorrhagic Fever Virus | NP | CCSE01P: TGTCAACACAGCAGGGTGCATGTAGAT  CCSE03P: TGTAAGCACGGCAGGGTGCATGTAAAT  CCSE0AP:  ACTCCAATGAAGTGGGGGAAGAAGCT | CCSE01F: CAAGGGGTACCAAGAAAATGAAGAAGGC | CCSE02R:  GCCACAGGGATTGTTCCAAAGCAGAC | [1] |
| Ebolavirus (Sudan/Zaire) | GP* | GP1DZ_P: CTACCAGCAGCGCCAGACGGGA  GP1DS_P: TTACCCCCACCGCCRGATGGT | EboGP_F: ATGGGCTGAAAAYTGCTACAATC | EboGP_R: CTTTGTGMACATASCGGCAC | [2] |
| Tai Forest ebolavirus | L | CCCATTACCGCAGATC | TTCATTAGTTGAAATCAAAACCGG | GAGAGTACAGTTATACATTGATTGTC | In-house |
| Bundibugyo ebolavirus | NP* | ACAAATCCAAGTGCACGC | CCTGTCTGGAGAAGGTTCAACG | TCGGATATTGAATCAGACCTTGTTC | [3] |
| Lassa virus | GPC |  | ACCGGGGATCCTAGGCATTT | GTTCTTTGTGCAGGARAGRGGCATRGTCAT | [4] |
| Marburg virus | VP40 | AAATTGCTCATRATCCCRAGAGGCAGCCA | GCGTATAACGARCGAACAGTCA  GCTTATAATGAGCGGACGGTCA | AGCCACAGTATGRGCTAAAGGRTATTC | In-house |
| Rift Valley Fever Virus | S | CAAACTCTCGGACCCAC | CTTGGCATCCTTCTCCCAGTC | ATCCAGTTTGCTGCTCAAGCA | In-house |

*with minor modifications to published assay

References

[1] R. Wölfel, J.T. Paweska, N. Petersen, A.A. Grobbelaar, P.A. Leman, R. Hewson, M.-C. Georges-Courbot, A. Papa, S. Günther, C. Drosten, Virus detection and monitoring of viral load in Crimean-Congo hemorrhagic fever virus patients, Emerging Infect. Dis. 13 (2007) 1097–1100. https://doi.org/10.3201/eid1307.070068.

[2] T.R. Gibb, D.A. Norwood, N. Woollen, E.A. Henchal, Development and evaluation of a fluorogenic 5’ nuclease assay to detect and differentiate between Ebola virus subtypes Zaire and Sudan, J. Clin. Microbiol. 39 (2001) 4125–4130. https://doi.org/10.1128/JCM.39.11.4125-4130.2001.

[3] J.S. Towner, T.K. Sealy, M.L. Khristova, C.G. Albariño, S. Conlan, S.A. Reeder, P.-L. Quan, W.I. Lipkin, R. Downing, J.W. Tappero, S. Okware, J. Lutwama, B. Bakamutumaho, J. Kayiwa, J.A. Comer, P.E. Rollin, T.G. Ksiazek, S.T. Nichol, Newly discovered ebola virus associated with hemorrhagic fever outbreak in Uganda, PLoS Pathog. 4 (2008) e1000212. https://doi.org/10.1371/journal.ppat.1000212.

[4] S. Olschläger, M. Lelke, P. Emmerich, M. Panning, C. Drosten, M. Hass, D. Asogun, D. Ehichioya, S. Omilabu, S. Günther, Improved detection of Lassa virus by reverse transcription-PCR targeting the 5’ region of S RNA, J. Clin. Microbiol. 48 (2010) 2009–2013. https://doi.org/10.1128/JCM.02351-09.
