# Supplementary material for: Retrospective meta-transcriptomic identification of severe dengue in a traveller returning from Africa to Sweden, 1990
Source: One Health. 2021 Jan 22;12:100217. doi: 10.1016/j.onehlt.2021.100217 (PMC7851179; doi:10.1016/j.onehlt.2021.100217)
Supplement: Supplementary Table S2 — Summary of clinical symptoms and findings. [file mmc2.docx]

Supplementary table S2. Summary of clinical symptoms [1] and findings.

| **Calender dates January–February 1990** | 17 | 18 | 19 | 20 | 21 | 22 | 23 | 24 | 25 | 26 | 27 | 28 | 29 | 30 | 31 | 1 | 2 | 3 | 4 | 5 | 6 | 7 | 8 | 9 | 10 | 11 | 12 | 13 | 14 | 15 | 16 | 17 | 18 | 19 | 20 | 21 | 22 |
| --- | --- | --- | --- | --- | --- | --- | --- | --- | --- | --- | --- | --- | --- | --- | --- | --- | --- | --- | --- | --- | --- | --- | --- | --- | --- | --- | --- | --- | --- | --- | --- | --- | --- | --- | --- | --- | --- |
| **Days in relation to symptom onset** | 0 | 1 | 2 | 3 | 4 | 5 | 6 | 7 | 8 | 9 | 10 | 11 | 12 | 13 | 14 | 15 | 16 | 17 | 18 | 19 | 20 | 21 | 22 | 23 | 24 | 25 | 26 | 27 | 28 | 29 | 30 | 31 | 32 | 33 | 34 | 35 | 36 |
| Urine samples |  |  |  |  |  |  |  |  |  |  |  |  |  |  |  |  |  |  |  |  |  |  |  |  |  |  |  |  | X |  |  |  | X |  |  |  | X |
| Plasma samples |  |  |  |  |  |  |  | X |  |  | X | X |  | X | X | X | X |  | X |  |  |  |  |  |  |  |  |  |  |  |  |  |  |  |  |  |  |
| Dengue detected with RNAseq |  |  |  |  |  |  |  | X |  |  |  |  |  | X | X | X | X |  | X |  |  |  |  |  |  |  |  |  |  |  |  |  |  |  |  |  |  |
| HPgV detected with RNAseq |  |  |  |  |  |  |  | X |  |  |  |  |  | X | X |  |  |  |  |  |  |  |  |  |  |  |  |  |  |  |  |  |  |  |  |  |  |
| Dengue detected with real-time PCR |  |  |  |  |  |  |  | X |  |  | X | X |  | X | X | X | X |  | X |  |  |  |  |  |  |  |  |  |  |  |  |  |  |  |  |  |  |
| Dengue serology (IgM/IgG) |  |  |  |  |  |  |  |  |  |  | X | X |  | X |  |  |  |  |  |  |  |  |  |  |  |  |  |  |  |  |  |  |  |  |  |  |  |
| Presentation with fever, headache, chills | X |  |  |  |  |  |  |  |  |  |  |  |  |  |  |  |  |  |  |  |  |  |  |  |  |  |  |  |  |  |  |  |  |  |  |  |  |
| Presentation with high fever and diarrhoea |  |  |  | X |  |  |  |  |  |  |  |  |  |  |  |  |  |  |  |  |  |  |  |  |  |  |  |  |  |  |  |  |  |  |  |  |  |
| Admitted to infectious disease ward |  |  |  |  | X | X | X |  |  |  |  |  |  |  |  |  |  |  |  |  |  |  |  |  |  |  |  |  |  |  |  |  |  |  |  |  |  |
| Fever above 40C |  |  |  | X | X | X | X | X | X | X | X | X | X | X | X | X | X | X | X | X | X | X | X |  |  |  |  |  |  |  |  |  |  |  |  |  |  |
| Transfer to ICU* |  |  |  |  |  |  | / | X | X | X | X | X | X | X | X | X | X | X | X | X | X | X | X | X | X | X | X | X | X | X | X | X |  |  |  |  |  |
| Haemorrhagic episode** |  |  |  |  |  |  | / | X | X | X | X | X | X | X | X | X | X | X | X | X | X | X |  |  |  |  |  |  |  |  |  |  |  |  |  |  |  |
| Respiratory treatment |  |  |  |  |  |  |  |  |  | X | X | X | X | X | X | X | X | X | X | X | X | X |  |  |  |  |  |  |  |  |  |  |  |  |  |  |  |
| Respiratory treatment weaning period |  |  |  |  |  |  |  |  |  |  |  |  |  |  |  |  |  |  |  |  |  |  | X | X | X | X |  |  |  |  |  |  |  |  |  |  |  |
| Septicaemia |  |  |  |  |  |  |  |  |  |  |  |  |  |  |  |  |  |  |  |  |  |  |  |  |  |  | X | X |  |  |  |  |  |  |  |  |  |
| Transfer from ICU to ward |  |  |  |  |  |  |  |  |  |  |  |  |  |  |  |  |  |  |  |  |  |  |  |  |  |  |  |  |  |  |  |  | X | X | X | X | X |
| * start on the 23rd or 24th of January 1990 | |  |  |  |  |  |  |  |  |  |  |  |  |  |  |  |  |  |  |  |  |  |  |  |  |  |  |  |  |  |  |  |  |  |  |  |  |

[1] U. Foberg, A. Frydén, B. Isaksson, P. Jahrling, A. Johnson, K. McKee, B. Niklasson, B. Normann, C. Peters, M. Bengtsson, Viral haemorrhagic fever in Sweden: experiences from management of a case, Scand. J. Infect. Dis. 23 (1991) 143–151. https://doi.org/10.3109/00365549109023392.
